# Production of Mycelium Mats for Textile Applications

**DOI:** 10.3390/jof11100700

**Published:** 2025-09-26

**Authors:** Reyes K. Romero-Cedillo, Efrén R. Robledo-Leal, Liliana Aguilar-Marcelino, Ma. de Lourdes Acosta-Urdapilleta, Maura Téllez-Téllez

**Affiliations:** 1 Maestría en Manejo de Recursos Naturales, Universidad Autónoma del Estado de Morelos, Cuernavaca 62209, Morelos, Mexico; reyes19septiembre@gmail.com; 2 Centro de Investigaciones Biológicas, Universidad Autónoma del Estado de Morelos, Av. Universidad No. 1001, Col. Chamilpa, Cuernavaca 62209, Morelos, Mexico; urdapilletal@yahoo.com; 3 Facultad de Ciencias Biológicas, Universidad Autónoma de Nuevo León, Monterrey 66455, Nuevo León, Mexico; efren.robledoll@uanl.edu.mx; 4 Centro Nacional de Investigación Disciplinaria en Salud Animal e Inocuidad (CENID-SAI) del INIFAP, Jiutepec 62550, Morelos, Mexico; aguilar.liliana@inifap.gob.mx

**Keywords:** Basidiomycete, liquid culture, mycelium material, *Trametes versicolor*, jute

## Abstract

A mycelium is a network of hyphae that possesses the ability to self-assemble and grow into various shapes, acting as a natural binder that minimises the need for intensive chemical and energy processes, making it an alternative capable of forming structures that may eventually outperform traditional fibres such as animal leather and polyester. In this work, two mycelium mats were created, and their thickness, water absorption, coverage, and tear strength for the sewing process were determined. Fibre mats were grown in vitro or on a jute substrate. The mats were treated with salt, tannin or citric acid solutions, then air- or oven-dried. In general, the treatment that least modified the colour and appearance of the mycelium mats was citric acid, and when dried by airflow, the thickness averaged 1.4 mm. The highest tear strengths were 10.55 N/mm and 12.7 N/mm for the mycelium mats treated with citric acid without and with jute, respectively. A high percentage of water absorption was observed, reaching 267% (mycelium mats treated with tannins and dried at 65 °C) and 28% (mycelium mats treated with citric acid and air-dried). In general, all mycelium mats can be sewn, except for those treated with citric acid, which have a viscous texture and require slow sewing to prevent the mycelium from breaking. The *Trametes* fungus can be utilised in the production of mycelial materials, allowing for the optimisation of growth conditions to obtain mycelial mats that meet the requirements for use as an environmentally friendly alternative in the textile and related industries.

## 1. Introduction

Bioconversion in an anthropogenic context has generated growing interest in the development of bio-based production processes for creating natural materials. A notable innovation arising from this trend is the growing number of patent applications that utilise a fungal mycelium as a raw material for producing conventional, low-energy materials, exploiting the natural growth of fungi within the production process [1].

Nowadays, the fashion industry faces significant challenges, particularly with respect to mitigating environmental pollution. The production of animal-derived leather raises critical concerns regarding animal welfare, ecological degradation, and potential risks to human health, primarily attributable to the toxic chemicals employed in tanning and processing. In response to these health-related risks, numerous countries have implemented stringent regulations or, in some cases, prohibitions on specific leather-processing practices [2]. Synthetic leather, typically manufactured from plastic-based polymers, introduces distinct environmental concerns. During use, washing, and disposal, these materials release microplastic particles [3], which exhibit limited biodegradability and consequently persist in ecosystems, contributing to long-term environmental pollution [4]. These limitations underscore the need to develop biologically inspired alternatives that minimise environmental and human health hazards. Recent research has therefore focused on materials with low ecological impact across their life cycle, from resource extraction to production and eventual disposal [5]. Fungal mycelium-based materials are particularly promising in this regard owing to their environmentally benign production processes and inherent biodegradability at the end of their service life.

Fungal-derived compounds are gaining wide acceptance due to their biodegradability, cost-effectiveness and novelty. Among these materials, those made using mycelium stand out for their ability to form networks of hyphae that bind organic matter through natural biological processes. The technological improvements being made during processing allow manufacturers to customise mycelium material to meet specific structural and functional requirements, including impact resistance, fire retardancy, and thermal and acoustic insulation [6]. The growth structure of fungi is the mycelium, which is responsible for nutrient uptake and colonisation. A mycelium is the result of an interconnected system of hyphae, which present a multilayered structure composed of glucans, mannoproteins, chitosan, chitin, polyglucuronic acid and small amounts of proteins and glycoproteins [7].

Among the biomaterials derived from fungal mycelia are mycelium-based foams (MBFs), which are grown on agricultural waste; mycelium-based sandwich composites (MBSCs), in which outer layers are made that surround a central core using natural fibres [8]; and pure mycelial materials (PMMs), which are composed solely of mycelium [9]. This technology generates sustainable materials with adaptable characteristics, such as foam, paper, and leather, that exhibit polymer-like properties [10]. PMMs have versatile characteristics and could replace petrochemical or animal-derived polymeric leather [11].

The properties of fungal-based materials can be tuned by introducing additives during their growth period; technical and aesthetic properties can be diversified through minimal variations in the manufacturing process, such as substrate composition or the fungal species used, and through appropriate post-growth treatment [12,13]. It has also been reported that materials obtained from monomitic mycelia have lower structural integrity than those from dimitic and trimitic systems. The trimitic system of *Trametes versicolor* has shown higher tensile and flexural strength than the monomitic system of *Pleurotus ostreatus* when grown on rapeseed straw [7]. Therefore, the objective of this work was to develop a mycelium mat and evaluate whether their physical and mechanical attributes are suitable for textile applications.

## 2. Materials and Methods

### 2.1. Biological Material

The *Trametes versicolor* strain (HEMIM-170), maintained at the Mycological Herbarium of Morelos (Autonomous University of the State of Morelos), was cultured on potato dextrose agar (PDA) for 5 days prior to use as an inoculum.

### 2.2. Growth Conditions

The growth medium was prepared with the following composition (DIFCO; g/L): glucose (10), corn starch (5), sucrose (5), yeast extract (1), malt extract (1), peptone (1), MgSO_4_ (0.3), KH_2_PO_4_·H_2_O (0.8), and K_2_HPO_4_ (0.2); it was sterilised by autoclaving at 15 psi for 15 min.

A jute fabric (Cosmos brand México, 280 mm × 190 mm; density 1.44 g/m^3^; areal weight 73 gr/m^2^; rough burlap texture) was washed three times with neutral detergent, thoroughly rinsed with running water to remove all detergent residues, and then sterilised. Once prepared, the jute was placed in trays for mycelium growth along with the culture medium.

The inoculum was prepared in 250 mL flasks containing 100 mL of the culture medium. Three mycelial fragments (4 mm in diameter) were added to each flask and incubated at 25 °C for 7 days on an orbital shaker at 130 rpm. After this period, the inoculum was transferred to trays (300 mm × 205 mm) containing the sterile culture medium, with or without jute support. The trays were incubated at 25 °C in the dark under static conditions until the mycelium fully colonised the surface (approximately 20 days). Two types of mycelium mats were produced: (1) mycelium and (2) mycelium with jute support.

### 2.3. Treatment of Mycelium Mats

After growth, the mycelial mats were removed from the trays and rinsed with water to remove excess culture broth. The mycelial mats were placed for 1 h in different solutions: (A) potassium aluminium sulphate dodecahydrate (aluminium potassium sulphate; 10%; room temperature), (B) tannins (procyanidins, catechin and epicatechin; 10%; room temperature), and (C) citric acid (10%; 90 °C). Subsequently, they were placed in glycerin (40%; 2 h) and dried using two methods: (a) air drying in a cabinet with an extractor and (b) drying at 65 °C.

### 2.4. Characterisation of Mycelium Mats

#### 2.4.1. Thickness Determination

The average thickness is reported. The measurement was taken under humid conditions and after the sample was dried. Each sample was placed on a flat surface and measured with a digital vernier calliper (Mitutoyo LCD150, Naucalpan de Juárez, Mexico). The thickness of two zones per side was recorded, resulting in a total of 8 measurements per sample. The analysis was performed in triplicate.

#### 2.4.2. Tear Strength

Tear strength was determined according to ISO 9073-3 for textiles, with modifications [14], using the test method for nonwovens, part 3. The mycelium mats with and without support were cut into rectangular shapes (60 mm × 20 mm), and the tear strength was determined using a digital hand dynamometer (PROCONSA, model EFG2, Monterrey, Mexico), reported as force per unit thickness of each sample (N/mm). The analysis was performed in triplicate.

#### 2.4.3. Water Absorption

Initially, the dry weight of each mycelium mat (60 mm × 20 mm) was recorded; subsequently, they were immersed in distilled water for 24 h. After this time, they were placed on a grid to remove excess water and weighed to calculate the percentage of water absorption, as determined by the following formula [15]. The analysis was performed in triplicate.

$$\mathrm{Water}\;\mathrm{absorption}\;\left(\%\right)=\left(\begin{array}{c} \frac{\mathrm{wet}\;\mathrm{weight}-\mathrm{dry}\;\mathrm{weight}}{\mathrm{dry}}\\ weight \end{array}\right)\times 100$$

#### 2.4.4. Mycelial Density

The surface layer of the mycelium was observed using a stereoscopic microscope (Olympus U-TV with camera SZX16; Ciudad de Mexico, Mexico). It was confirmed that the mats formed a homogeneous layer of mycelium and colour along the length and width of the obtained material.

#### 2.4.5. Sewing

Each sample was sewn with a straight-edge seam using a Singer Facilita Pro (4432; México) machine with a lining fabric (Arletex Tenancingo, Mexico, 100% polyester; light and smooth texture). The analysis was performed in triplicate.

### 2.5. Statistical Analysis

The Shapiro normality test was performed. Subsequently, an ANOVA (Statistica® 14.0.0) was used to determine differences between the experimental data, and for those that showed significant differences, the Tukey test was performed.

## 3. Results

### 3.1. Obtaining Mycelium Mats

**Figure 1:**
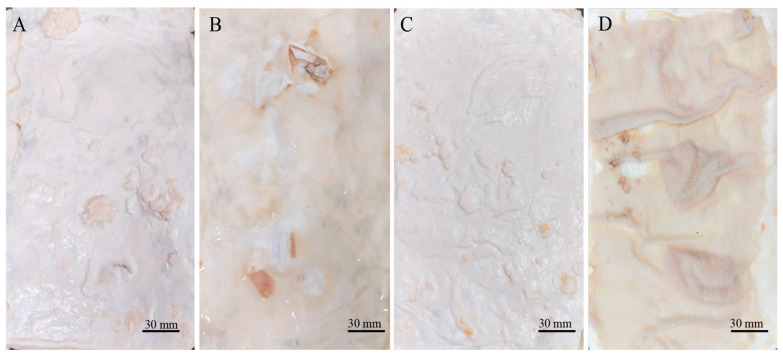
Appearance of moist mycelium mats formed by *T. versicolor*. (**A**) Mycelium mats for which the surface exposed to the air is observed; (**B**) mycelium mat is observed on the side in contact with the culture medium; (**C**) jute-supported mycelium mat, air-exposed surface; and (**D**) jute-supported mycelium mat, culture medium side.

Two types of mycelium mats were obtained. The wet appearance of the mycelium mats (Figure 1A) was white and velvety, displaying the typical texture of mycelia. In the case of the mycelium mats with support, they also exhibited a velvety texture, but the mycelium conformed to the shape of the jute (Figure 1C). In both cases, one side of the mycelium mats (which was exposed to the air) had a velvety appearance, while the opposite side had a gelatinous appearance with a light brown colour (Figure 1B,D).

### 3.2. Treatment of Mycelium Mats

**Figure 2:**
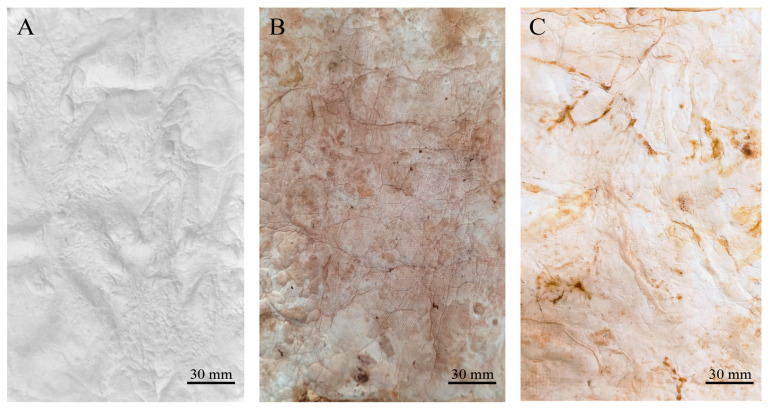
Mycelium mats using *T. versicolor* after treatments. (**A**) Mycelium exposed to air treated with aluminium potassium sulphate, (**B**) mycelium exposed to air treated with tannins, and (**C**) mycelium exposed to air treated with citric acid.

**Figure 3:**
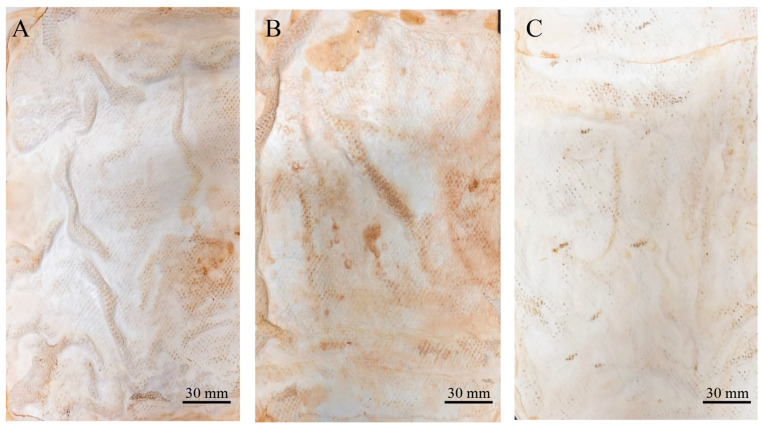
Supported mycelium mats using *T. versicolor* after treatments. (**A**) Mycelium exposed to air treated with aluminium potassium sulphate, (**B**) mycelium exposed to air treated with tannins, and (**C**) mycelium exposed to air treated with citric acid.

After submitting the materials to all treatments (treatment A, aluminium potassium sulphate; treatment B, tannins; treatment C, citric acid), treatment with aluminium potassium sulphate showed no visible changes, indicating that the salts did not affect the mycelium mats. In treatment with tannins, the mycelium mats acquired a pink–violet coloration due to the tannins. In contrast, treatment with citric acid resulted in a light brown colouration, with darker areas appearing in some regions (Figure 2). The mycelium mats with support developed a light brown colour in all three treatments, with some darker areas; however, this change was less noticeable in the citric acid treatment (Figure 3).

**Figure 4:**
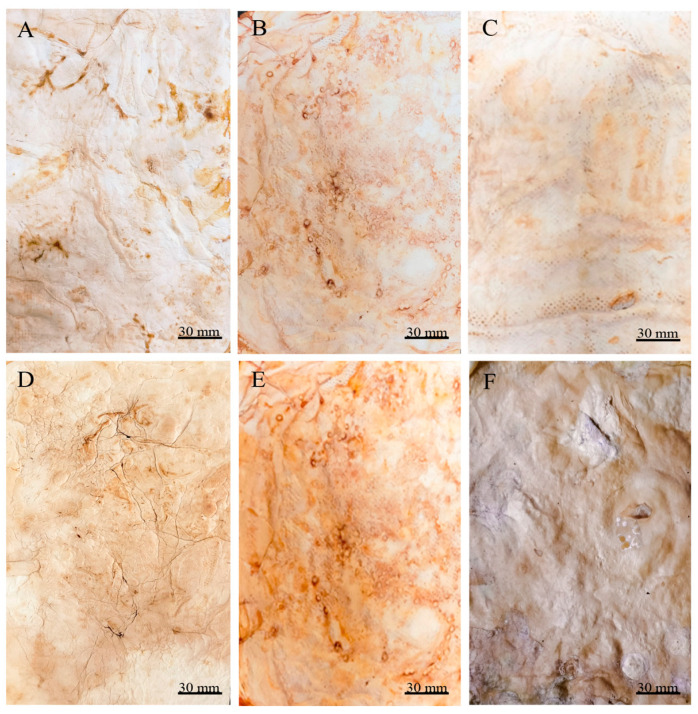
Mycelium mats produced using *Trametes versicolor* after chemical treatments and drying. (**A**) Air-dried + aluminium potassium sulphate, (**B**) air-dried + tannins, (**C**) air-dried + citric acid, (**D**) dried at 65 °C + aluminium potassium sulphate, (**E**) dried at 65 °C + tannins, and (**F**) dried at 65 °C + citric acid.

**Figure 5:**
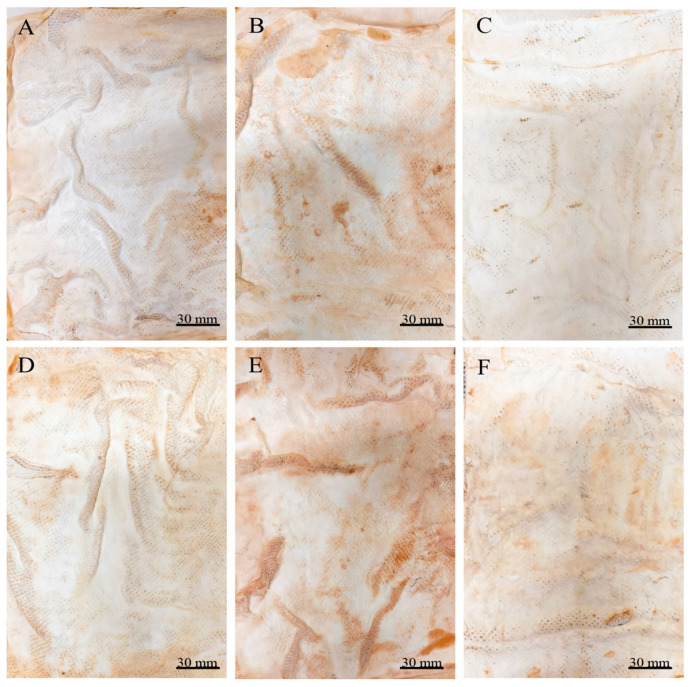
Supported mycelium mats produced using *Trametes versicolor* after chemical treatments and drying. (**A**) Air-dried + aluminium potassium sulphate, (**B**) air-dried + tannins, (**C**) air-dried + citric acid, (**D**) dried at 65 °C + aluminium potassium sulphate, (**E**) dried at 65 °C + tannins, and (**F**) dried at 65 °C + citric acid.

Both drying methods resulted in the proportional shrinkage of each side of the mycelial mats due to water loss (Figure 4); however, air drying resulted in less noticeable changes in the appearance of the mycelium mats (Figure 4A–C). In contrast, drying at 65 °C resulted in more pronounced alterations in appearance and a more intense colouration (Figure 4D–F). For the jute-supported fibre, the colour change was less noticeable under both treatments (Figure 5), with the air-dried samples showing minimal alteration in appearance (Figure 5A–C).

### 3.3. Characterisation of the Mycelium Mats

#### 3.3.1. Thickness

**Figure 6:**
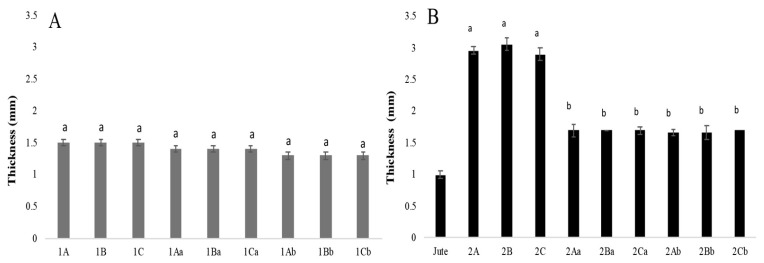
Mycelium mat thickness of *T. versicolor*. (**A**) Mycelium mats and (**B**) mycelium mats with jute. The means share the same letter, indicating that they are not statistically different according to Tukey (*p* < 0.05).

The wet mycelial mats after each treatment (aluminium and potassium sulphate, tannins, and citric acid) had a thickness of 1.5 mm. After air drying, the thickness was 1.4 mm (tannins and citric acid), and after drying at 65 °C, the thickness was 1.3 mm, except for the mycelial mats treated with citric acid, which measured 1.4 mm and showed no statistical differences (Figure 6A). The mycelial mats with jute had an initial wet thickness of 2.9 mm in all three treatments. After drying at 65 °C, the thickness was 1.7 mm. After air drying, the aluminium potassium sulphate and tannin-treated samples were 1.6 mm thick, and the citric acid-treated sample was 1.7 mm thick. Considering the jute layer itself was 1 mm thick after being washed and sterilised (Figure 6B), both types of mats lost approximately 0.2 mm of mycelial thickness regardless of the drying method used.

#### 3.3.2. Tear Strength

**Figure 7:**
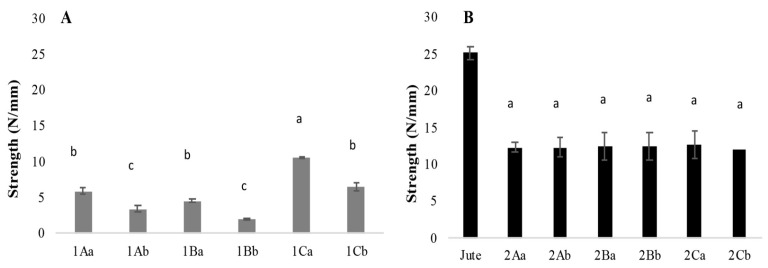
Tear strength of mycelium textile fibres. (**A**) Mycelium and (**B**) mycelium with jute. The means share the same letter, indicating that they are not statistically different according to Tukey (*p* < 0.05).

In the samples without support, a statistically significant difference in tear strength was observed between treatments. The highest tear strength was recorded in the air-dried sample treated with citric acid, reaching 10.55 N/mm, whereas the same treatment dried at 65 °C showed a strength of 6.55 N/mm. The lowest tear strength was observed in the samples treated with tannins using the heat drying method, with a value of 1.9 N/mm compared with 4.6 N/mm for the air-dried samples. These results suggest that drying at elevated temperatures negatively affects the tear strength of unsupported mycelium mats (Figure 7A).

For the mycelium mats with jute support, no significant differences in tear strength were found across treatments or drying methods. The treatment with citric acid of air-dried samples reached 12.78 N/mm, while the heat-dried counterparts demonstrated a tear strength of 12.1 N/mm. However, since the tear strength of the washed and sterilised jute was 25 N/mm, the results indicate that the growth of mycelium on the support weakens its overall strength (Figure 7B).

#### 3.3.3. Water Absorption

**Figure 8:**
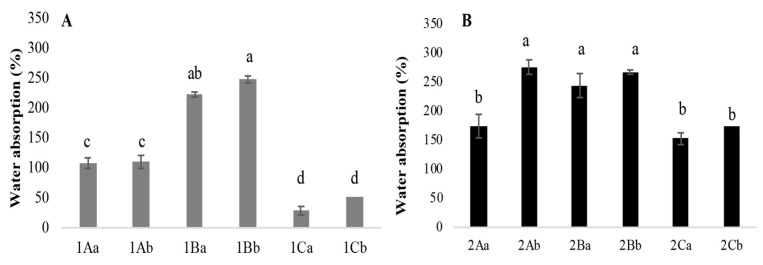
Percentage of water absorption of mycelium mats. (**A**) Mycelium mats and (**B**) mycelium mats with jute. The means share the same letter, indicating that they are not statistically different according to Tukey (*p* < 0.05).

The mycelium mats absorbed between 28% and 267% of water, with the jute-supported fibres showing a higher absorption overall. Among the different treatments, the mycelium mats treated with tannins and dried at 65 °C exhibited the highest water absorption. In contrast, the mycelium mats treated with citric acid and air-dried showed the lowest (Figure 8). The mycelium mats treated with aluminium potassium sulphate did not show statistically significant differences between drying methods, with water absorption values of 108% (air drying) and 110% (65 °C) (Figure 8A). Ashraf et al. [16] indicated that the hydroxyl groups present in the cellulose chain of jute interact with other hydroxyl groups and water molecules. In addition, the hydrogen bonds between these hydroxyl groups are also responsible for their hydrophilic nature; for this reason, the mycelium mats with jute presented greater water absorption.

#### 3.3.4. Mycelial Density

**Figure 9:**
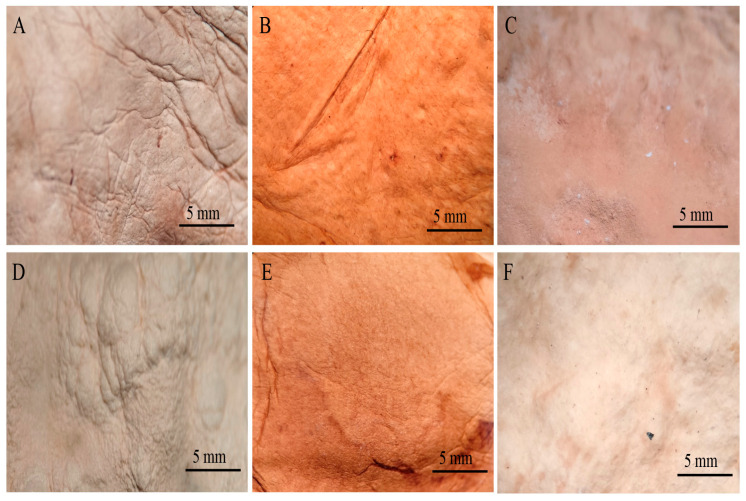
Mycelium mats of *T. versicolor* observed under a stereomicroscope. (**A**) Air-dried + aluminium potassium sulphate, (**B**) air-dried + tannins, (**C**) air-dried + citric acid, (**D**) dried at 65 °C + aluminium potassium sulphate, (**E**) dried at 65 °C + tannins, and (**F**) dried at 65 °C + citric acid.

By observing the mycelial mats under a microscope, the surface appearance and colour of each of the mycelial mats were qualitatively evaluated. In general, all mycelium mats exhibited folds, with their visibility varying depending on the treatment. The mycelium mats treated with aluminium potassium sulphate showed no significant colour change, while the most noticeable colour modification occurred in the tannin-treated samples. In the samples treated with citric acid and air-dried, more fold surfaces were observed. For the mycelium mats dried at 65 °C, those treated with aluminium potassium sulphate showed no visible changes, whereas the tannin- and citric acid-treated samples exhibited clear coloration (Figure 9).

**Figure 10:**
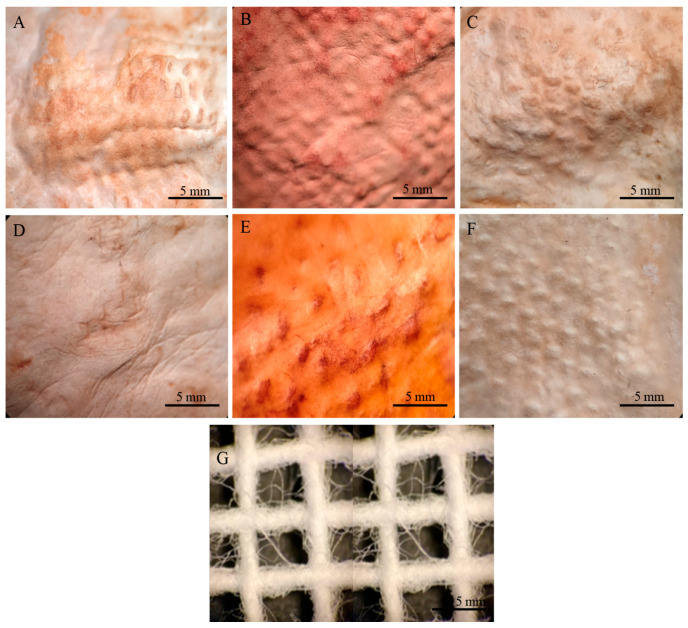
Mycelium mats of *T. versicolor* with jute observed under a stereomicroscope. (**A**) Air-dried + aluminium potassium sulphate, (**B**) air-dried + tannins, (**C**) air-dried + citric acid, (**D**) dried at 65 °C + aluminium potassium sulphate, (**E**) dried at 65 °C + tannins, and (**F**) dried at 65 °C + citric acid. (**G**) Jute.

In the case of the mycelium mats with jute support, the structural changes of the mycelium mats were visible to the naked eye, with the mycelium conforming to the texture of the support. These mycelium mats presented similar folds to the unsupported mycelium mats. Mycelial growth was evident across the jute surface, with hyphae filling the gaps in the supporting material (Figure 10).

#### 3.3.5. Sewing Performance

**Figure 11:**
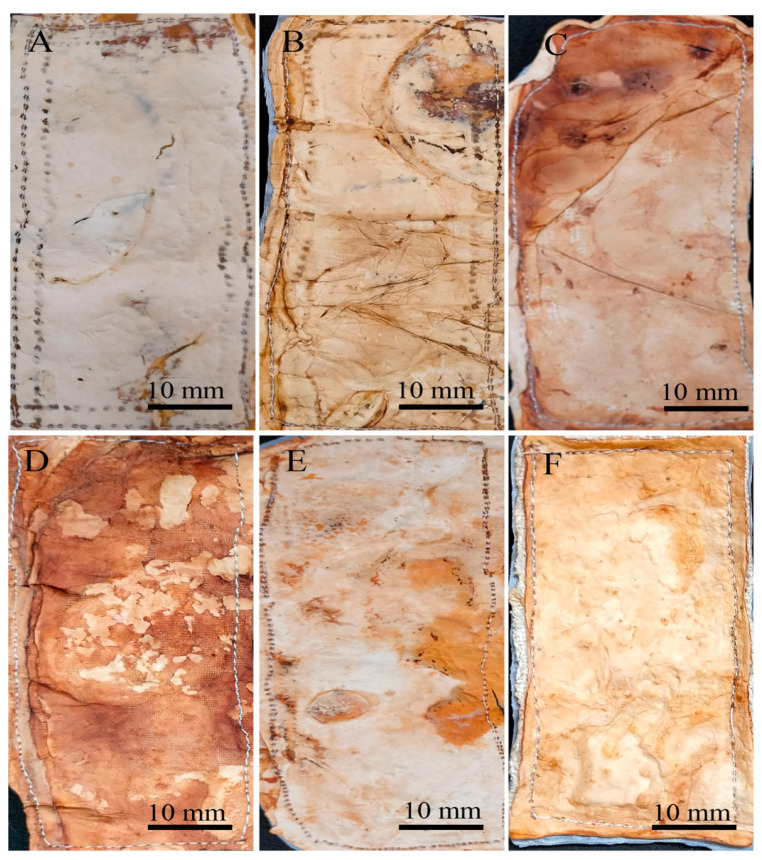
Mycelium mats of *T. versicolor* after the stitching process. (**A**) Air-dried aluminium potassium sulphate, (**B**) air-dried tannins, (**C**) air-dried citric acid, (**D**) aluminium potassium sulphate dried at 65 °C, (**E**) tannins dried at 65 °C, and (**F**) citric acid dried at 65 °C.

**Figure 12:**
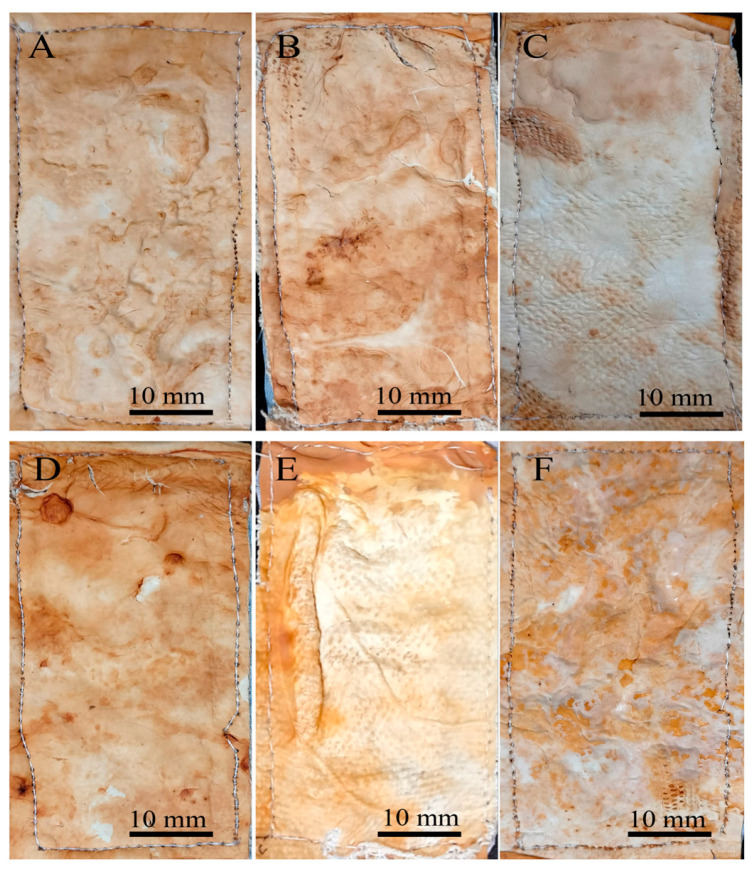
Mycelium mats with jute of *T. versicolor* after the stitching process. (**A**) Air-dried aluminium potassium sulphate, (**B**) air-dried tannins, (**C**) air-dried citric acid, (**D**) aluminium potassium sulphate dried at 65 °C, (**E**) tannins dried at 65 °C, and (**F**) citric acid dried at 65 °C.

A sewing test was conducted considering that sewing is a critical step in leather and textile processing that enables their use in a wide range of accessories. During the sewing process, the mycelium mats underwent a noticeable change in appearance, with all samples acquiring a dark brown colour. When touching or squeezing the mycelium, some compounds are oxidised and this coloration appears (Figure 11 and Figure 12). Overall, the mats were handled without major problems during sewing. However, difficulties were encountered when treated with citric acid (Figure 11E,F and Figure 12E,F). In these cases, the sewing machine needle tended to jam, requiring greater care and time to avoid tearing or breaking the mycelial structure. The rest of the treatments did not exhibit those difficulties during sewing.

## 4. Discussion

Fungal materials are generally classified into pure fungal materials and composites [17]. Among the latter are mycelium-based sandwich composites (MBSCs), which consist of a mycelial core covered by two or more outer layers. Lignocellulosic fibres or natural textiles, such as linen, jute, or cellulose, have been used to form these outer layers, which are shaped according to the desired final product [18]. The characteristics of these composites depend on several factors, including the substrate, fungal species, and growth conditions [19,20,21]. In this research, both unsupported mycelium mats and mycelium mats with support were produced using a liquid culture method. Previous studies have indicated that semi-solid and liquid culture systems are suitable for producing high-density, flexible, and mechanically robust mycelial materials [22].

An essential factor in preparing the material is the culture medium and/or substrates used, as they can enhance the growth and productivity of the mycelium. To improve the application of fungal material, it is essential to optimise and control mycelial growth, as well as to employ biochemical tools related to the structure of fungal growth, since the mechanical characteristics and texture are of great interest [23]. Bae et al. [22] used 64 Polyporales strains to characterise the mycelium; they indicated that all strains showed good mycelial growth, but most formed a mycelial mat of low physical resistance except for the *Ganoderma lucidum* strain, which exhibited mycelium with excellent physical resistance that was attributed to its thick hyphae, with a diameter of 13 μm, while the hyphae of other organisms showed a diameter less than 2 μm.

Cartabia et al. [24] reported that mats formed by *Trametes* species were fragile and inconsistent, *Ganoderma carnosum* and *Ganoderma lucidum* failed to form uniform mats, *Fomitopsis pinicola* formed dense but brittle mats, and *Fomitopsis iberica* (*Fomitopsis marianiae*; Index Fungorum) [25] and *Fomitopsis mediterranea* produced consistent and homogeneous mats showing aerial mycelium; however, this last species grows very slowly. The dry weight of the mycelial mats was variably affected by the substrate residues embedded in the mycelium, but such bias was lower when the mat was more consistent, as in the case of *Fomes fomentarius* and *Irpiciporus pachyodon*. Raman et al. [7] reported that they obtained dense mycelia from 14 fungal strains, except for *Elfvingia applanata* (*Ganoderma applanatum*; Index Fungorum) [25] and *Trametes hirsuta*; in the cases of *Ganoderma lucidum*, *Ganoderma applanatum*, *Fomitella fraxinea*, *Fomitopsis pinicola*-KCTC and *Postia balsamea*, they obtained highly dense and flexible mycelial mats, and, finally, for *Fomitopsis pinicola*-JF (*Fomitopsis pinicola*; Index Fungorum) [25], *Fomitopsis rosea* (*Rhodofomes roseus*; Index Fungorum) [25], *Trametes versicolor*, *Trametes suaveolens*, *Wolfiporia extensa*, *Microporus affinis* and *Bjerkandera adusta*, they produced high-density mycelium, but these were very fragile, not flexible and of low tensile strength. In this work, flexible mycelial mats of *Trametes versicolor* were obtained. Those without jute were more flexible, but they lacked a uniform appearance. However, the inoculation and growth conditions can be optimised to achieve a more desirable appearance.

Girometta et al. [4] reported the use of flax to produce fungal materials, indicating that it resulted in greater biomass production and efficient colonisation. Overall, MBSCs exhibit heterogeneity between the laminated layers and the core; however, they are mechanically strong and rigid structures, displaying a higher modulus of elasticity and tensile strength than materials composed solely of mycelia. This is consistent with the results of this research, as the mycelium mats with jute showed significant tensile strength. Another characteristic of fungal materials is water absorption; *Pleurotus* mycelial composites grown on grain fibres have been reported to absorb up to 114–278% of their weight when submerged for 24 h, significantly increasing their weight [26].

Appels et al. [27] reported that *Trametes multicolor* (*Trametes ochracea*; Index Fungorum) [25] materials were coarser than those of *Pleurotus ostreatus*; *T. multicolor* (*Trametes ochracea*; Index Fungorum) [25] material increased in weight by 508% (unpressed rapeseed straw), 436% (hot-pressed rapeseed straw), and 43% (unpressed beech sawdust), while *P. ostreatus* material increased in weight by 274% (unpressed rapeseed straw) and 238% (hot-pressed cotton). Overall, unpressed and cold-pressed materials had a whitish, velvety appearance with a foam-like structure, and heat-pressed materials were compact and brown in appearance. Furthermore, individual hyphae within the mycelium were less visible after heat pressing due to hyphal adhesion to the substrate and a denser material compared with unpressed materials. In this work, the citric acid-treated mycelial mat was exposed to temperature (90 °C/1 h), which may have favoured hyphal adhesion and porosity modification, resulting in greater tear resistance (Figure 7) and lower water absorption (Figure 8).

During the drying process, the fungal materials can also be modified to improve the characteristics of the mycelium (colour, texture, appearance, flexibility, etc.), drying has been carried out through lyophilisation and/or vacuum filtration through membranes [28,29]. However, this requires equipment and infrastructure that are not widely available. Since drying in this study was performed using two methods (temperature (65 °C) and flowing air), it was observed that the mycelium mats’ appearance was more modified when exposed to heat. In this regard, Attias et al. [29] reported that semi-dry mycelium films can be transferred to a frame for forced drying to prevent shrinkage and wrinkling or alternatively placed on a flat surface and covered with cellophane [28]. In this work, the mycelium mats were placed on a smooth surface during the drying process to prevent them from forming folds. However, folds were observed in the wet mycelium mats, as mycelium clumps formed, resulting in the folds within the mycelium mats.

Another essential characteristic is mechanical tear strength. Moreno et al. [30] reported that paiche skin (*Arapaima gigas*) tanned with 10% quebracho (*Schinopsis balansae*) and 10% mimosa (*Acacia dealbata*) tannins achieved a tensile strength of 14.94 N/mm^2^, meeting the requirements of the Peruvian Technical Standards NTP 241.021:2022 [31], NTP 241.022:2022 [32], and NTP 241.023:2022 [33], which specify a minimum tensile strength of ≥10 N/mm^2^ for the manufacture of men’s and women’s footwear. In the present study, mycelium mats with jute support treated with tannins and air-dried reached a tensile strength of 12.57 N/mm^2^, while the same treatment with heat drying resulted in 12.43 N/mm^2^ values, which also fall within the acceptable range for these textile applications. Jute is a biodegradable fibre that is composed of lignin (12–14%), hemicellulose (22–26%), cellulose (59–63%), fats, waxes and pectins [13], so the fungus, when growing on said support, can use it as a source of nutrients, since *Trametes* is a non-selective white rot fungus that degrades lignin, hemicellulose and cellulose at a similar rate [34]. We assume that this is why the strength of jute is reduced when obtaining the mycelium mat.

In some works, mycelium is treated to maintain its characteristics and reinforce it, as indicated by Jones et al. [35], who performed chemical and thermal treatments to make the material durable and resistant to environmental stress, as well as to preserve or improve its structural and appearance characteristics. Among these treatments, crosslinking agents (such as citric acid) and plasticisers (such as glycerol) are used either during or after growth [28,29]; it has been indicated that the characteristics are improved and the mycelial structure is reinforced. Raman et al. [7] obtained a highly dense material from the mycelium of *Fomitella fraxinea* grown on a solid substrate, which was subjected to plasticisation, crosslinking and surface coating. They observed that the coating with corn zein increased the yellowish colour of the fibre and gave it a glassy appearance; crosslinking with 5–10% tannic acid desirably altered the colour, improving the appearance. Those treated with 10% tannic acid displayed a dark reddish-yellow colour and strengthened mycelial structure.

Bentangan et al. [36] indicated that to preserve the mycelial tissue and prevent rot degradation, it can be boiled and treated with salt. In this study, the best treatment maintaining the appearance of the mycelial mat was with aluminium and potassium sulphate (mycelium) and citric acid (mycelium with jute). The mycelial mats treated with citric acid showed a lower water absorption and tear resistance. In this regard, it has been reported that citric acid reacts with the hydroxyl groups of glucans, causing crosslinking between the cell walls of the hyphae [37]. Additionally, it has been noted that, in certain systems, the crosslinking agent (citric acid) substantially increases the material’s viscosity by enhancing the molecular weight of the polymer chains [38]. Therefore, in this work, the material treated with citric acid presented a slightly viscous texture, so greater care was taken when carrying out the sewing process.

Aluminium potassium sulphate is used in artisanal leather tanning; it has been reported to provide durability, softness, colour, and flexibility [39]. In this connection, it is stated that tannins have weak hydrogen bonds, which is usually not sufficient to stabilise the structure of leather, accordingly reflected in a low thermal stability, and the salts favour cross-linking, which stabilises the collagen structure and increases the thermal stability of the leather [40]. In this work, with respect to tear resistance, there was no difference between mycelium mats treated with tannins and salts; in the case of water absorption, treatment with salts absorbed less water, so that interaction probably strengthened the stability of the mycelium mats. In general, the mycelium mats after treatment acquired a light to dark brown coloration (dried at 65 °C); this may have been related to what Appels et al. [27] reported, that the darkening of mycelium-based composites is due to Maillard reactions involving the sugars and proteins present in the cell walls of the fungi or the caramelisation of fungal sugars. The material of fungi does not mechanically resemble bovine leather, since it has low tear and tensile strength, as well as a high susceptibility to abrasion. It has been suggested that these properties are due to the high content of α-glucan, which can reach 53% of the dry weight [41]. Furthermore, it is essential to remember that Appels et al. [42] indicated that the mechanical properties of materials obtained using wild mushroom mycelia presented similar characteristics to those of plant materials (wood, cork, bamboo) and animal materials (leather), which is mainly due to an increase in density and not to greater tensile strength of the material. Therefore, it is crucial to continue analysing and obtaining materials from wild mushrooms, as different characteristics in relation to resistance, durability, and flexibility can be obtained depending on the type of mushroom and the cultivation system.

## 5. Conclusions

*Trametes versicolor* is a white-rot fungus with trimitic hyphal system that presents a high growth rate and biomass yield, and it is typically resistant to environmental stress, making it a viable candidate for industrial applications. Thin and flexible mycelium mats were obtained in this study; those treated with citric acid showed low water absorption and greater resistance, but they were not easy to handle, and the mycelium mats treated with salts presented low water absorbance, making them a feasible option in the textile industry. The mats with jute as support showed a greater resistance but a higher water absorption capacity, so it is possible to use them in some areas that are not exposed to direct contact with water, which should be evaluated.

## Data Availability

Data is contained within the article. The original contributions presented in this study are included in the article. Further inquiries can be directed to the corresponding author.

## Abbreviations

The following abbreviations are used in this manuscript:

1A	Mycelium mats + aluminium potassium sulphate
1B	Mycelium mats + tannins
1C	Mycelium mats + citric acid
1Aa	Mycelium mats + aluminium potassium sulphate +air-dried
1Ba	Mycelium mats + tannins + air-dried
1Ca	Mycelium mats + citric acid + air-dried
1Ab	Mycelium mats + aluminium potassium sulphate + dried at 65 °C
1Bb	Mycelium mats + tannins + dried at 65 °C
1Cb	Mycelium mats + citric acid + dried at 65 °C
2A	Supported mycelium mats + aluminium potassium sulphate
2B	Supported mycelium mats + tannins
2C	Supported mycelium mats + citric acid
2Aa	Supported mycelium mats + aluminium potassium sulphate + air-dried
2Ba	Supported mycelium mats + tannins + air-dried
2Ca	Supported mycelium mats + citric acid + air-dried
2Ab	Supported mycelium mats + aluminium potassium sulphate + dried at 65 °C
2Bb	Supported mycelium mats + tannins + dried at 65 °C
2Cb	Supported mycelium mats + citric acid + dried at 65 °C
HEMIM	Mycological Herbarium of Morelos
K_2_HPO_4_	Potassium phosphate dibasic
KH_2_PO_4_·H_2_O	Monopotassium phosphate monohydrate
MBSCs	Mycelium-based sandwich composites
MBFs	Mycelium-based foams
MgSO4	Magnesium sulphate
N/mm	Newton per millimetre
PMMs	Pure mycelial materials
PDA	Potato dextrose agar
